# Machine Learning and Metabolomics Predict Mesenchymal Stem Cell Osteogenic Differentiation in 2D and 3D Cultures

**DOI:** 10.3390/jfb15120367

**Published:** 2024-12-05

**Authors:** Michail E. Klontzas, Spyros I. Vernardis, Aristea Batsali, Fotios Papadogiannis, Nicki Panoskaltsis, Athanasios Mantalaris

**Affiliations:** 1 Artificial Intelligence and Translational Imaging (ATI) Lab, Department of Radiology, School of Medicine, University of Crete, 71003 Heraklion, Greece; 2 Computational Biomedicine Laboratory, Institute of Computer Science, Foundation for Research and Technology (ICS-FORTH), 70013 Heraklion, Greece; 3 The Francis Crick Institute, London NW1 1AT, UK; 4 Haemopoiesis Research Laboratory, School of Medicine, University of Crete, 71003 Heraklion, Greece; 5 BioMedical Systems Engineering Laboratory, Panoz Institute, School of Pharmacy and Pharmaceutical Sciences, Trinity College, D02 PN40 Dublin, Ireland; 6 National Institute for Bioprocessing Research and Training (NIBRT), Foster Avenue, Mount Merrion, Blackrock, A94 X099 Dublin, Ireland

**Keywords:** metabolomics, mesenchymal stem cells, machine learning, osteogenesis, differentiation, biomanufacturing

## Abstract

Stem cells have been widely used to produce artificial bone grafts. Nonetheless, the variability in the degree of stem cell differentiation is an inherent drawback of artificial graft development and requires robust evaluation tools that can certify the quality of stem cell-based products and avoid source-tissue-related and patient-specific variability in outcomes. Omics analyses have been utilised for the evaluation of stem cell attributes in all stages of stem cell biomanufacturing. Herein, metabolomics in combination with machine learning was utilised for the benchmarking of osteogenic differentiation quality in 2D and 3D cultures. Metabolomics analysis was performed with the use of gas chromatography–mass spectrometry (GC-MS). A set of 11 metabolites was used to train an XGboost model which achieved excellent performance in distinguishing between differentiated and undifferentiated umbilical cord blood mesenchymal stem cells (UCB MSCs). The model was benchmarked against samples not present in the training set, being able to efficiently capture osteogenesis in 3D UCB MSC cultures with an area under the curve (AUC) of 82.6%. On the contrary, the model did not capture any differentiation in Wharton’s Jelly MSC samples, which are well-known underperformers in osteogenic differentiation (AUC of 56.2%). Mineralisation was significantly correlated with the levels of fumarate, glycerol, and myo-inositol, the four metabolites found most important for model performance (R^2^ = 0.89, R^2^ = 0.94, and R^2^ = 0.96, and *p* = 0.016, *p* = 0.0059, and *p* = 0.0022, respectively). In conclusion, our results indicate that metabolomics in combination with machine learning can be used for the development of reliable potency assays for the evaluation of Advanced Therapy Medicinal Products.

## 1. Introduction

The limited availability of bone autografts and the associated donor site morbidity dictate the need for the development of artificial bone grafts which can overcome these shortcomings [1]. Stem cells and especially mesenchymal stem cells (MSCs) have been widely utilised for the production of artificial bone grafts, combined with a wide variety of biomaterials and stimulated with appropriate osteoinductive factors. However, the variability in the degree of stem cell differentiation in biomaterials is an inherent drawback of artificial graft development and requires robust evaluation tools that can certify the quality of stem cell-based products and avoid source-tissue-related and patient-specific variability in outcomes [2].

Advances in analytical tools and omics technologies have enabled the quantification and analysis of an unprecedented volume of molecular data. This plethora of information enables the development of sensitive and specific methods for the evaluation of culture quality for cell therapy applications. Complex omics data analysis has been utilised for the evaluation of stem cell attributes in all stages of stem cell biomanufacturing, the input, the bioprocess, and the output [3,4].

Metabolomics, as a profiling tool enabling the dynamic monitoring of physiological changes, allows the sensitive and robust characterisation of intracellular and extracellular phenotype [5]. Metabolomics has proven its usefulness to capture the changes in cellular physiology during stem cell differentiation. This has been utilised for the identification of differential effects of osteoinductive factors and for the monitoring of osteogenic differentiation quality in 3D environments such as hydrogels [2,6]. The ability to sensitively capture fine differences in stem cell physiology renders metabolomics a powerful tool for the quality control of stem cell manufacturing.

Machine learning (ML) can be used as a powerful method to construct classifiers that predict stem cell physiology. NMR metabolomics has been used to assess metabolic alterations during adipogenic differentiation [7] and to predict stem cell potency by means of metabolite fingerprinting and footprinting [8]. These models have not probed stem cell differentiation towards the osteogenic lineage which is of utmost importance for the production of engineered bone grafts [9] and have been developed for 2D cultures which are not commonly used in biomanufacturing.

**Figure 1:**
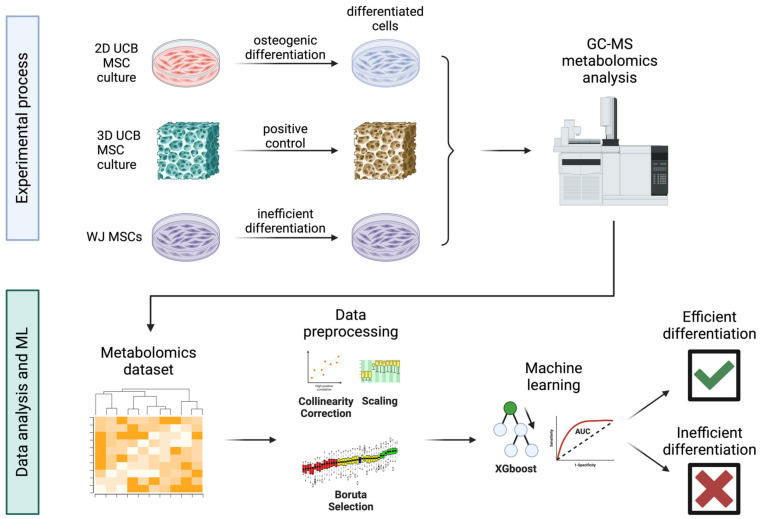
Flowchart describing the experimental and machine learning pipeline (created with biorender.com).

Herein, we present an ML pipeline which employs intracellular metabolite data for the benchmarking of osteogenic differentiation quality in 2D and 3D cultures. Umbilical cord blood mesenchymal stem cells (UCB MSCs) have been used to train machine learning models of osteogenic differentiation (Figure 1). These models were trained to distinguish between differentiated and undifferentiated cells in 2D cultures and were benchmarked against well-differentiated 3D cultures and poorly differentiated Wharton’s jelly MSC 2D cultures. The proposed ML pipeline captures cellular differentiation and can be used in cell therapy manufacturing.

## 2. Materials and Methods

### 2.1. Cell Isolation and Culture

MSCs were isolated from full-term cord blood units (NHS Blood and Transplant, Colindale Branch, London, UK) and umbilical cord tissue (Public Cord Blood Bank, Heraklion, Greece) according to the relevant ethical approvals with the consent of the mothers. WJ MSCs and UCB MSCs were isolated with the explant method and Ficoll density gradient centrifugation as previously described and expanded with the use of α-ΜΕΜ Glutamax-I 10% FBS and 1% penicillin/streptomycin under 5% CO_2_, 21% O_2_, and 37 °C. Cells at passage 5–6 were used for further experiments. Osteogenic differentiation was induced with the supplementation of basal medium with 10^−7^ M dexamethasone, 10 mM β-glycerophosphate, and 50 μg/mL of ascorbic acid 2-phosphate for 21 days. Data for Alizarin Red S (ARS) quantification and collagen 1 A1 protein levels were obtained from prior work [10] and correlated with metabolite levels.

### 2.2. Three-Dimensional Polyurethane Scaffold Fabrication and Functionalization

Three-dimensional porous polyurethane scaffolds were prepared by means of thermally induced phase separation. Briefly, 3 g of polyurethane was dissolved in 60 mL of 1,4-dioxane at 80 °C and frozen at −80 °C, and the solvent was removed by sublimation in a polyethylene glycol bath (−15 °C), covalently functionalized with RGD according to a published protocol. Scaffolds were functionalized with a solution of 100 μg/mL (R100) of RGD and compared to non-functionalized polyurethane scaffolds (Con) as previously described [11].

### 2.3. GC-MS Metabolomics Analysis

Metabolomics analysis was performed according to prior publications [2,12]. Briefly, metabolites were extracted from 2D cultures of UCB and Wharton’s jelly MSCs by quenching the metabolism with ice-cold methanol, following a water/methanol (50:50) extraction protocol as described by Kanani et al. [12,13].

A similar extraction procedure was followed for UCB MSCs cultured on polyurethane scaffolds, which were washed with PBS, soaked in 3 mL of ice-cold methanol for 5 min, and repeatedly squeezed to ensure optimal metabolite extraction from cells seeded in the scaffolds. Internal standards were added relative to cell numbers calculated by means of DNA quantification (1 μg of ribitol and 2 μg of UC13-glucose per 10^6^ cells) as previously described [2]. Dry polar metabolite extracts were derivatized with the use of 50 μL of methoxyamine hydrochloride for 1.5 h followed by 100 μL of N-Methyl-N-(trimethylsilyl) trifluoroacetamide for 6 h and ran in quadruplicate injections in a Shimadzu QP2010 Ultra gas chromatography–mass spectrometry machine [2,14]. Metabolomics data for the 2D osteogenesis of UCB MSCs were obtained from previously published work [10].

### 2.4. Data Pre-Processing and Feature Selection

Data standardisation, normalisation, and filtering was performed according to Kanani et al. [13]. Relative peak areas were calculated with respect to the peak area of ribitol, and metabolites with a coefficient of variation <30% over the quadruplicate GC-MS runs were excluded from the analysis. Similarly, peaks with a signal-to-noise ratio lower than 3 and all unknown metabolites were removed from further biomarker analysis.

Collinearity correction was performed to remove highly correlated redundant features with a Pearson correlation coefficient >80%. Subsequently, Boruta feature selection was applied to select the most relevant features for machine learning model development. Boruta is a tree-based machine learning algorithm used to select informative features for model development [15].

### 2.5. Machine Learning Model Development

Metabolomics data from 2D UCB MSC cultures were used to train an XGboost gradient boosting machine learning model to predict whether cells are in a differentiated (d21) or undifferentiated state. For this purpose, 2D culture data were randomly split 70:30% into training/testing sets using a random seed. The model was built with 5-fold cross-validation in the training set and hyperparameters were optimised with the use of random search for 1000 rounds. The model was trained with early stopping at n = 30 epochs to avoid overfitting by monitoring the log-loss of the model in the training step. Features important for XGboost classification were calculated for the trained model.

The model was then externally validated using a positive control dataset where osteogenic differentiation has been confirmed (3D UCB MSC) [11] and a negative control dataset (WJ MSCs) of cells well known to underperform in osteogenic differentiation compared to UCB MSCs [16,17]. All cells were differentiated with the same protocol. The process of model development is demonstrated on Figure S1.

### 2.6. Statistics

Descriptive statistics are presented with the use of frequencies, and summary statistics are presented as mean ± standard deviation.

Model performance was assessed with the use of areas under the curve (AUCs) of receiver operating characteristic (ROC) curves with the respective 95% confidence intervals. Statistics were performed with the use of R v4.2.2 (www.R-project.org, accessed on 3 December 2024) and machine learning was performed with the caret, XGboost, pROC, and e1071 packages. Boruta feature selection was implemented with the Boruta package. Sensitivity, specificity, and positive and negative predictive values were also calculated. Statistical significance was defined with a *p*-value less than a = 0.05.

## 3. Results

**Figure 2:**
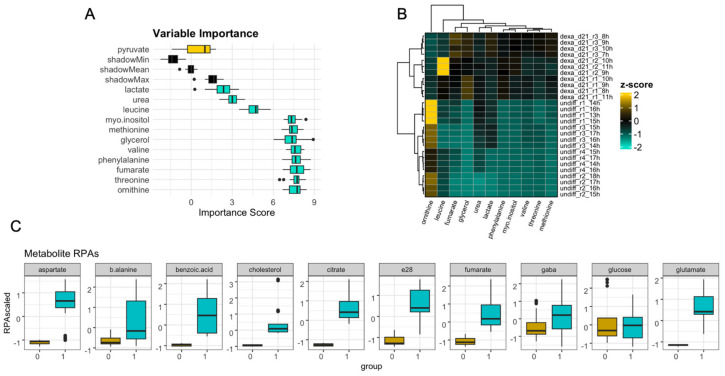
Metabolomics variables important for model predictions. Variable importance analysis (**A**) indicates metabolites selected for model training with the use of Boruta feature selection analysis. The levels of these metabolites in undifferentiated and differentiated cells were analysed with hierarchical clustering (Euclidean distance) (**B**), and boxplots (**C**) indicating a wide variety of differences in metabolite abundance between the two groups.

A total of 35 metabolites were used for machine learning model building. Collinearity correction and Boruta feature selection yielded 11 metabolites for use in model training (Figure 2A). Unsupervised hierarchical clustering was performed to identify potential relationships between the selected metabolites across undifferentiated and differentiated samples. All metabolites clustered together apart from ornithine which clustered independently (Figure 2B,C). Importantly, clustering of the samples based on the containing metabolites showed that undifferentiated cells formed a separate cluster compared to differentiated cells, indicating systematic differences in metabolite levels between the two cell states. A variable degree of difference between relative peak areas (RPAs) of the selected metabolites was shown between the undifferentiated samples and samples on day 21 of osteogenesis (Figure 2C with the majority of metabolites exhibiting a higher relative peak area in differentiated compared to undifferentiated samples.

**Figure 3:**
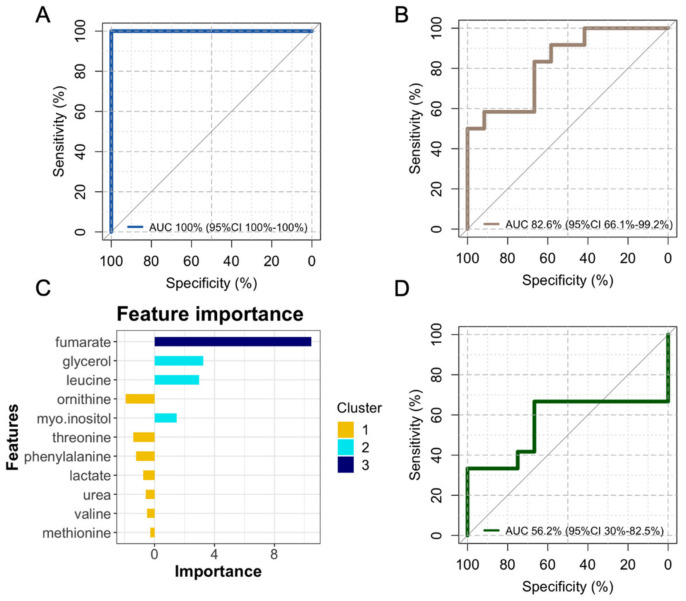
Machine learning model performance analysis. Receiver operating characteristic (ROC) curves of 2D (**A**) and 3D (**B**) umbilical cord blood mesenchymal stem cell (MSC) cultures indicate excellent areas under the curve (AUCs) and the respective 95% confidence intervals. Feature importance analysis for model performance indicates the effect of specific metabolites on model results (**C**), and the low performance of the model on negative-control Wharton’s jelly MSC cultures is indicated in the respective ROC graph (**D**).

An XGboost model was trained using UCB MSCs differentiated towards the osteoblastic lineage in 2D cultures. The model achieved an AUC of 100% when internally tested with an unseen hold-out UCB MSC test set (Figure 3A). To assess the performance of the model in different conditions and assess model performance drift in external data, the model was tested with a positive control of metabolomics data from 3D cultures with confirmed efficient differentiation [11]. When tested in 3D cultures, the model displayed a performance drift of 17.4%. Nonetheless, despite the performance drift, the model still achieved a good performance (AUC of 82.6%, 95%CI of 66.1–99.2%) in the positive control group (Figure 3B), indicating that even when externally tested, it can sufficiently predict the degree of differentiation of probed samples.

To further demonstrate the efficiency of the model, negative control testing was performed with the use of metabolomics data from WJ MSCs, which are known to underperform in osteogenic differentiation. In the case of WJ MSCs, the model failed to capture differentiation, demonstrating an AUC close to a random guess (AUC 56.2%, 95%CI 30–82.5%) (Figure 3D).

Factor importance analysis on the trained XGboost model indicated that the factor with the biggest importance on model prediction was fumarate followed by a cluster of factors positively affecting model predictions (glycerol, leucine, myo-inositol), whereas the remaining metabolites had a negative importance on model predictions (Figure 3C).

**Figure 4:**
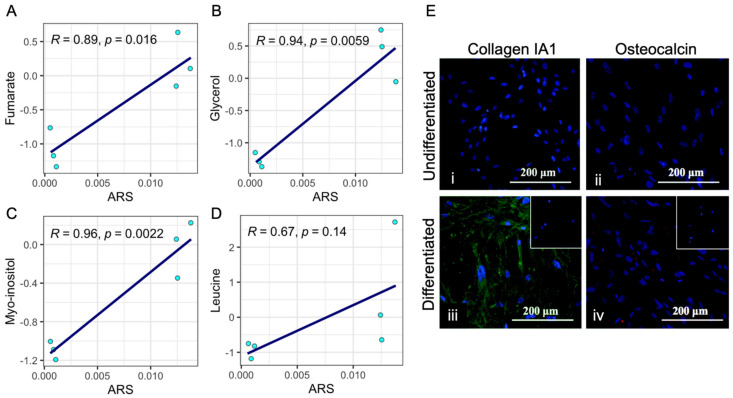
Correlation between metabolite features found important for machine learning predictions, and the culture mineralisation. Levels of metabolites with a positive influence on model performance (fumarate—(**A**); glycerol—(**B**); myo-inositol—(**C**); leucine—(**D**)) were correlated with Alizarin Red S quantification results. The expression of osteocalcin and collagen I A1 in differentiated cultures is demonstrated in ((**E**), (**i**,**iii**) represent Collagen I A1 levels in undifferentiated and differentiated cultures respectively, (**ii**,**iv**) represent osteocalcin levels in undifferentiated and differentiated cultures respectively) (Alizarin Red S and immunofluorescence data were adapted with permission ref. [10]).

In order to assess the relationship between metabolites with a positive impact on model prediction and the efficiency of differentiation, the mineralization of differentiated and undifferentiated cultures was assessed with the use of ARS staining and quantification. As indicated by ARS quantification, the degree of culture mineralization was correlated with the level of the four metabolites with the greatest positive impact on model prediction, as indicated by the aforementioned importance analysis (fumarate, glycerol, myo-inositol, leucine) (Figure 3C). Indeed, ARS levels significantly correlated with the levels of all the metabolites: fumarate, glycerol, and myo-inositol (R^2^ = 0.89, R^2^ = 0.94, and R^2^ = 0.96, and *p* = 0.016, *p* = 0.0059, and *p* = 0.0022, respectively) (Figure 4A–D). Mature osteogenic differentiation in the cultures used for model training had been confirmed by the identification of collagen 1 A1 and osteocalcin by means of immunofluorescence analysis. As indicated by immunofluorescence, both osteocalcin and collagen I A1 (markers of terminal differentiation) were expressed in differentiated samples but were not detected in undifferentiated cultures (Figure 4E).

## 4. Discussion

Herein, machine learning was utilised to predict the differentiation stage of UCB MSCs with the use of global metabolomics signatures. Importantly, machine learning models trained with metabolomics data could predict the stage of osteogenic differentiation in 2D and 3D cultures. The algorithm also captured the inefficient differentiation of WJ MSCs, predicting that after 21 days of differentiation, cells remained similar to the undifferentiated group. Our study is the first to utilise machine learning-driven metabolomics to evaluate the quality of osteogenic differentiation of MSCs.

Machine learning using 2D metabolomics data sufficiently captured the degree of differentiation in 3D cultures. As previously shown, UCB MSCs achieve efficient differentiation towards the osteogenic lineage in 3D PU scaffolds functionalised with RGD [11]. The transition from the 2D to 3D culture environment is known to reduce the proliferation capacity of MSCs while driving the cells towards differentiation [18]. These changes are accompanied by distinct alterations of their metabolic phenotype including the reduced activity of glycolysis, glutaminolysis, and the TCA cycle. We have previously shown that metabolomics can efficiently capture all these metabolic alterations accompanying stem cell proliferation and differentiation [2,10]. The methodology proposed herein exploits the capacity of metabolomics to capture the degree of differentiation and the ability of machine learning for automated predictions, to offer a tool for the identification of efficiently differentiated 2D and 3D MSC cultures. Future research could further validate the levels of metabolites significant for model performance using targeted approaches, as a step towards the development of targeted metabolism-driven assays for the monitoring of MSC cultures.

Machine learning failed to assign WJ MSCs to either the differentiated or the undifferentiated group. This result corroborates current knowledge that WJ MSCs do not efficiently differentiate towards the osteogenic lineage. There is strong evidence that WJ MSCs cannot exhibit terminal osteogenic differentiation unless exposed to bone marrow stimuli [19] or growth factors such as WNT1-inducible-signalling pathway protein 1 (WISP1) [16,20]. The poor performance of native WJ MSCs in osteogenic differentiation renders them suitable as a negative control in methods aiming to capture high-quality terminal osteogenesis. In our case, the model developed in 2D cultures was able to capture osteogenesis in a 3D setting but underperformed with WJ MSCs as a negative control, indicating the suitability of the model to detect both efficient and inefficient differentiation.

Being able to identify well-differentiated cells is important for biomanufacturing purposes. Omics-based assays can be used for the quality assurance and quality control (QA/QC) procedures necessary in the biomanufacturing of cell therapy products [21,22]. Metabolomics analysis can identify differentiated cells in 2D and 3D cultures and pinpoint biomarkers of differentiation [2,6,10]. We propose a method which can efficiently identify high-quality differentiated cells and can be used as a potency/release assay as required for all Advanced Therapy Medicinal Products (ATMPs) in the final stage of their manufacturing process [23,24,25,26,27,28]. Given the dynamic nature of metabolism and the complexity of metabolomics analysis [29,30,31,32], machine learning can revolutionise ATMP manufacturing, enabling the development of assays that utilise limited metabolite sets which indicate the quality of stem cell differentiation [33,34,35].

Model accuracy in 2D cultures was perfect, which could be potentially attributed to overfitting of the model due to the relatively small sample size [36,37]. Nonetheless, the performance of the method was also excellent when tested in independent 3D cultures, indicating that the results were representative and not attributable to overfitting. Measures to avoid overfitting such as early stopping were also taken during model training to ensure robust model performance [37,38,39]. These methods included testing on an independent subgroup of samples and the use of early stopping during model training. Testing in an external cohort from another lab could be a future step for the validation of our results [40,41,42,43].

In conclusion, our study demonstrates the effective application of machine learning and metabolomics to predict the differentiation stage of UCB MSCs, offering a robust method for evaluating osteogenic differentiation in both 2D and 3D cultures. The successful prediction of differentiation stages and the identification of inefficient differentiation in WJ MSCs underscore the potential of this approach for quality assurance in cell therapy biomanufacturing. While the model showed perfect accuracy in 2D cultures, its strong performance in 3D cultures indicates robustness and generalizability. These findings highlight the utility of metabolomics and machine learning in developing reliable potency assays for Advanced Therapy Medicinal Products (ATMPs).

## Data Availability

The original contributions presented in the study are included in the article, further inquiries can be directed to the corresponding authors.

## Supplementary Materials

The following supporting information can be downloaded at: https://www.mdpi.com/article/10.3390/jfb15120367/s1.

## References

[1] Dimitriou R., Jones E., McGonagle D., Giannoudis P.V. (2011). Bone Regeneration: Current Concepts and Future Directions. BMC Med..

[2] Klontzas M.E., Reakasame S., Silva R., Morais J.C.F., Vernardis S., MacFarlane R.J., Heliotis M., Tsiridis E., Panoskaltsis N., Boccaccini A.R. (2019). Oxidized Alginate Hydrogels with the GHK Peptide Enhance Cord Blood Mesenchymal Stem Cell Osteogenesis: A Paradigm for Metabolomics-Based Evaluation of Biomaterial Design. Acta Biomater..

[3] Lipsitz Y.Y., Timmins N.E., Zandstra P.W. (2016). Quality Cell Therapy Manufacturing by Design. Nat. Biotechnol..

[4] Salazar-Noratto G.E., Luo G., Denoeud C., Padrona M., Moya A., Bensidhoum M., Bizios R., Potier E., Logeart-Avramoglou D., Petite H. (2020). Understanding and Leveraging Cell Metabolism to Enhance Mesenchymal Stem Cell Transplantation Survival in Tissue Engineering and Regenerative Medicine Applications. Stem Cells.

[5] Cuperlović-Culf M., Barnett D.A., Culf A.S., Chute I. (2010). Cell Culture Metabolomics: Applications and Future Directions. Drug Discov. Today.

[6] Alakpa E.V., Jayawarna V., Lampel A., Burgess K.V., West C.C., Bakker S.C.J., Roy S., Javid N., Fleming S., Lamprou D.A. (2016). Tunable Supramolecular Hydrogels for Selection of Lineage-Guiding Metabolites in Stem Cell Cultures. Chem.

[7] Migdadi L., Sharar N., Jafar H., Telfah A., Hergenröder R., Wöhler C. (2023). Machine Learning in Automated Monitoring of Metabolic Changes Accompanying the Differentiation of Adipose-Tissue-Derived Human Mesenchymal Stem Cells Employing 1H-1H TOCSY NMR. Metabolites.

[8] Van Grouw A., Colonna M.B., Maughon T.S., Shen X., Larey A.M., Moore S.G., Yeago C., Fernández F.M., Edison A.S., Stice S.L. (2023). Development of a Robust Consensus Modeling Approach for Identifying Cellular and Media Metabolites Predictive of Mesenchymal Stromal Cell Potency. Stem Cells.

[9] De Long W.G., Einhorn T.A., Koval K., McKee M., Smith W., Sanders R., Watson T. (2007). Bone Grafts and Bone Graft Substitutes in Orthopaedic Trauma Surgery. A Critical Analysis. J. Bone Jt. Surg. Am..

[10] Klontzas M.E., Vernardis S.I., Heliotis M., Tsiridis E., Mantalaris A. (2017). Metabolomics Analysis of the Osteogenic Differentiation of Umbilical Cord Blood Mesenchymal Stem Cells Reveals Differential Sensitivity to Osteogenic Agents. Stem Cells Dev..

[11] Tahlawi A., Klontzas M.E., Allenby M.C., Morais J.C.F., Panoskaltsis N., Mantalaris A. (2019). RGD-Functionalized Polyurethane Scaffolds Promote Umbilical Cord Blood Mesenchymal Stem Cell Expansion and Osteogenic Differentiation. J. Tissue Eng. Regen. Med..

[12] Kanani H., Chrysanthopoulos P.K., Klapa M.I. (2008). Standardizing GC–MS Metabolomics. J. Chromatogr. B.

[13] Kanani H.H., Klapa M.I. (2007). Data Correction Strategy for Metabolomics Analysis Using Gas Chromatography-Mass Spectrometry. Metab. Eng..

[14] Vernardis S.I., Terzoudis K., Panoskaltsis N., Mantalaris A. (2017). Human Embryonic and Induced Pluripotent Stem Cells Maintain Phenotype but Alter Their Metabolism after Exposure to ROCK Inhibitor. Sci. Rep..

[15] Kursa M.B., Rudnicki W.R. (2010). Feature Selection with the Boruta Package. J. Stat. Softw..

[16] Batsali A.K., Pontikoglou C., Koutroulakis D., Pavlaki K.I., Damianaki A., Mavroudi I., Alpantaki K., Kouvidi E., Kontakis G., Papadaki H.A. (2017). Differential Expression of Cell Cycle and WNT Pathway-Related Genes Accounts for Differences in the Growth and Differentiation Potential of Wharton’s Jelly and Bone Marrow-Derived Mesenchymal Stem Cells. Stem Cell Res. Ther..

[17] Bosch J., Houben A.P., Radke T.F., Stapelkamp D., Bünemann E., Balan P., Buchheiser A., Liedtke S., Kögler G. (2012). Distinct Differentiation Potential of “MSC” Derived from Cord Blood and Umbilical Cord: Are Cord-Derived Cells True Mesenchymal Stromal Cells?. Stem Cells Dev..

[18] Rybkowska P., Radoszkiewicz K., Kawalec M., Dymkowska D., Zabłocka B., Zabłocki K., Sarnowska A. (2023). The Metabolic Changes between Monolayer (2D) and Three-Dimensional (3D) Culture Conditions in Human Mesenchymal Stem/Stromal Cells Derived from Adipose Tissue. Cells.

[19] Cabrera-Pérez R., Monguió-Tortajada M., Gámez-Valero A., Rojas-Márquez R., Borràs F.E., Roura S., Vives J. (2019). Osteogenic Commitment of Wharton’s Jelly Mesenchymal Stromal Cells: Mechanisms and Implications for Bioprocess Development and Clinical Application. Stem Cell Res. Ther..

[20] Xu L., Corcoran R.B., Welsh J.W., Pennica D., Levine A.J. (2000). WISP-1 Is a Wnt-1- and Beta-Catenin-Responsive Oncogene. Genes Dev..

[21] Lewis A.M., Abu-Absi N.R., Borys M.C., Li Z.J. (2016). The Use of ’Omics Technology to Rationally Improve Industrial Mammalian Cell Line Performance. Biotechnol. Bioeng..

[22] Samoudi M., Masson H.O., Kuo C.-C., Robinson C.M., Lewis N.E. (2021). From Omics to Cellular Mechanisms in Mammalian Cell Factory Development. Curr. Opin. Chem. Eng..

[23] Capelli C., Cuofano C., Pavoni C., Frigerio S., Lisini D., Nava S., Quaroni M., Colombo V., Galli F., Bezukladova S. (2023). Potency Assays and Biomarkers for Cell-Based Advanced Therapy Medicinal Products. Front. Immunol..

[24] Silva D.N., Chrobok M., Ahlén G., Blomberg P., Sällberg M., Pasetto A. (2022). ATMP development and pre-GMP environment in academia: A safety net for early cell and gene therapy development and manufacturing. Immunooncol. Technol..

[25] Manzini P., Peli V., Rivera-Ordaz A., Budelli S., Barilani M., Lazzari L. (2022). Validation of an automated cell counting method for cGMP manufacturing of human induced pluripotent stem cells. Biotechnol. Rep..

[26] Radrizzani M., Soncin S., Lo Cicero V., Andriolo G., Bolis S., Turchetto L. (2016). Quality Control Assays for Clinical-Grade Human Mesenchymal Stromal Cells: Methods for ATMP Release. Methods Mol. Biol..

[27] Lechanteur C., Briquet A., Bettonville V., Baudoux E., Beguin Y. (2021). MSC Manufacturing for Academic Clinical Trials: From a Clinical-Grade to a Full GMP-Compliant Process. Cells.

[28] Radrizzani M., Soncin S., Bolis S., Lo Cicero V., Andriolo G., Turchetto L. (2016). Quality Control Assays for Clinical-Grade Human Mesenchymal Stromal Cells: Validation Strategy. Methods Mol. Biol..

[29] Wang G., Heijs B., Kostidis S., Mahfouz A., Rietjens R.G.J., Bijkerk R., Koudijs A., van der Pluijm L.A.K., van den Berg C.W., Dumas S.J. (2022). Analyzing cell-type-specific dynamics of metabolism in kidney repair. Nat. Metab..

[30] Barberi G., Benedetti A., Diaz-Fernandez P., Sévin D.C., Vappiani J., Finka G., Bezzo F., Barolo M., Facco P. (2022). Integrating metabolome dynamics and process data to guide cell line selection in biopharmaceutical process development. Metab. Eng..

[31] Fiehn O. (2002). Metabolomics—The link between genotypes and phenotypes. Plant Mol. Biol..

[32] Miller I.J., Peters S.R., Overmyer K.A., Paulson B.R., Westphall M.S., Coon J.J. (2019). Real-time health monitoring through urine metabolomics. NPJ Digit Med..

[33] Ludikhuize M.C., Rodríguez Colman M.J. (2021). Metabolic Regulation of Stem Cells and Differentiation: A Forkhead Box O Transcription Factor Perspective. Antioxid. Redox Signal..

[34] Shyh-Chang N., Ng H.H. (2017). The metabolic programming of stem cells. Genes Dev..

[35] Leibel S.L., Tseu I., Zhou A., Hodges A., Yin J., Bilodeau C., Goltsis O., Post M. (2022). Metabolomic profiling of human pluripotent stem cell differentiation into lung progenitors. iScience.

[36] Rajput D., Wang W.J., Chen C.C. (2023). Evaluation of a decided sample size in machine learning applications. BMC Bioinform..

[37] Dhiman P., Ma J., Qi C., Bullock G., Sergeant J.C., Riley R.D., Collins G.S. (2023). Sample size requirements are not being considered in studies developing prediction models for binary outcomes: A systematic review. BMC Med. Res. Methodol..

[38] Prechelt L. (1998). Automatic early stopping using cross validation: Quantifying the criteria. Neural Netw..

[39] Zur R.M., Jiang Y., Pesce L.L., Drukker K. (2009). Noise injection for training artificial neural networks: A comparison with weight decay and early stopping. Med. Phys..

[40] Kernbach J.M., Staartjes V.E. (2022). Foundations of Machine Learning-Based Clinical Prediction Modeling: Part II-Generalization and Overfitting. Acta Neurochir. Suppl..

[41] Snell K.I.E., Allotey J., Smuk M., Hooper R., Chan C., Ahmed A., Chappell L.C., Von Dadelszen P., Green M., Kenny L. (2020). External validation of prognostic models predicting pre-eclampsia: Individual participant data meta-analysis. BMC Med..

[42] Collins G.S., Moons K.G.M., Dhiman P., Riley R.D., Beam A.L., Van Calster B., Ghassemi M., Liu X., Reitsma J.B., van Smeden M. (2024). TRIPOD+AI statement: Updated guidance for reporting clinical prediction models that use regression or machine learning methods. BMJ.

[43] Stevens L.M., Mortazavi B.J., Deo R.C., Curtis L., Kao D.P. (2020). Recommendations for Reporting Machine Learning Analyses in Clinical Research. Circ. Cardiovasc. Qual. Outcomes.

