# Supplementary figures and images for: Machine Learning and Metabolomics Predict Mesenchymal Stem Cell Osteogenic Differentiation in 2D and 3D Cultures
Source: J Funct Biomater. 2024 Dec 5;15(12):367. doi: 10.3390/jfb15120367 (PMC11680063; doi:10.3390/jfb15120367)

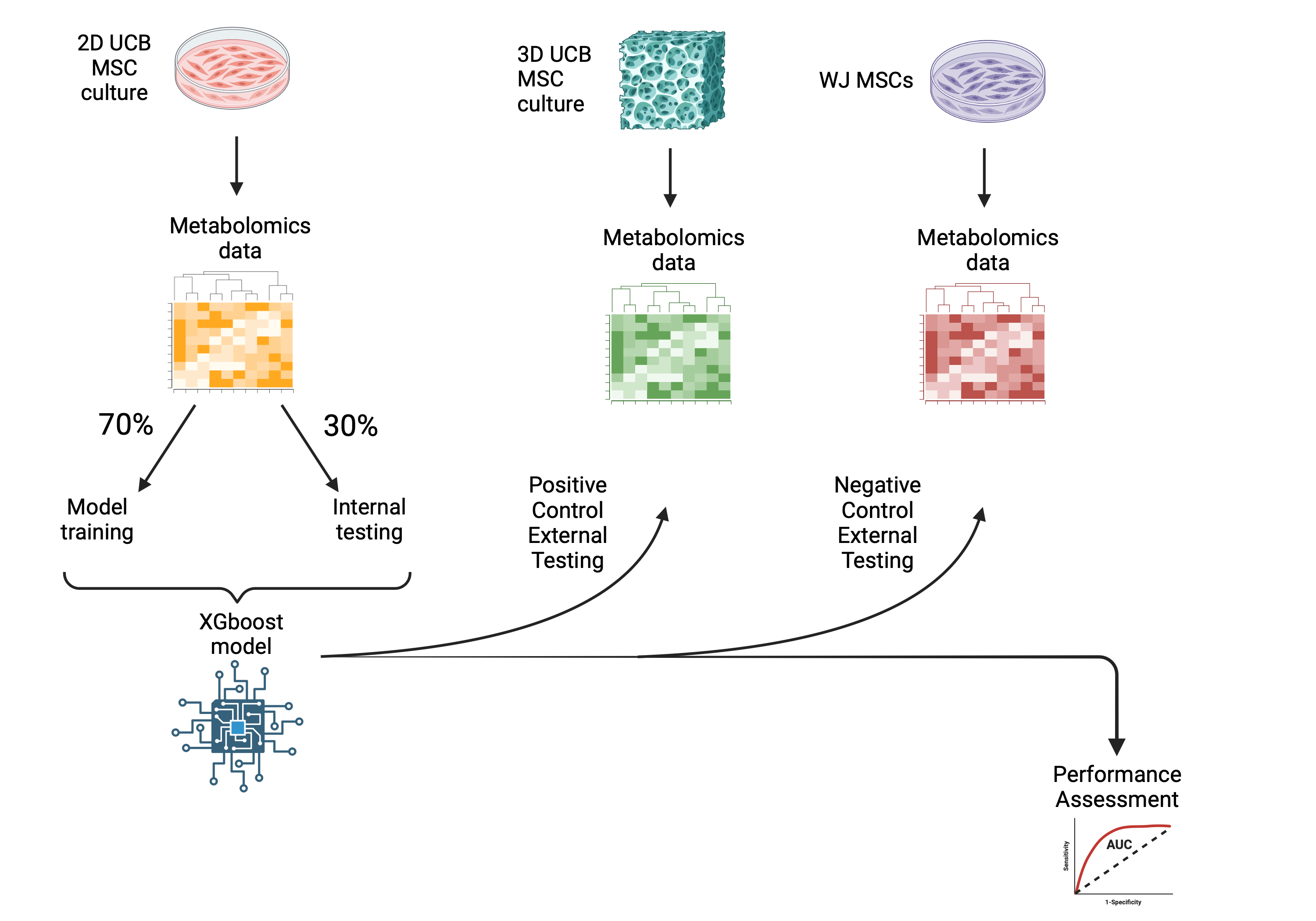

Supplement: Supplementary file 1 [file jfb-15-00367-s001.zip › Figure S1.png]
